# Volatile-mediated between-plant communication in Scots pine and the effects of elevated ozone

**DOI:** 10.1098/rspb.2022.0963

**Published:** 2022-09-14

**Authors:** Hao Yu, Minna Kivimäenpää, James D. Blande

**Affiliations:** Department of Environmental and Biological Sciences, University of Eastern Finland, PO Box 1627, 70211 Kuopio, Finland

**Keywords:** between-plant communication, ozone, photosynthesis, resin duct, Scots pine, volatile emissions

## Abstract

Conifers are dominant tree species in boreal forests, but are susceptible to attack by bark beetles. Upon bark beetle attack, conifers release substantial quantities of volatile organic compounds known as herbivore-induced plant volatiles (HIPVs). Earlier studies of broadleaved plants have shown that HIPVs provide information to neighbouring plants, which may enhance their defences. However, the defence responses of HIPV-receiver plants have not been described for conifers. Here we advance knowledge of plant–plant communication in conifers by documenting a suite of receiver-plant responses to bark-feeding-induced volatiles. Scots pine seedlings exposed to HIPVs were more resistant to subsequent weevil feeding and received less damage. Receiver plants had both induced and primed volatile emissions and their resin ducts had an increased epithelial cell (EC) mean area and an increased number of cells located in the second EC layer. Importantly, HIPV exposure increased stomatal conductance and net photosynthesis rate of receiver plants. Receiver-plant responses were also examined under elevated ozone conditions and found to be significantly altered. However, the final defence outcome was not affected. These findings demonstrate that HIPVs modulate conifer metabolism through responses spanning photosynthesis and chemical defence. The responses are adjusted under ozone stress, but the defence benefits remain intact.

## 1. Introduction

Plants constitutively emit volatile organic compounds (VOCs), but emissions can increase dramatically in response to stress [1–3]. VOCs induced by herbivore feeding and oviposition are commonly referred to as herbivore-induced plant volatiles (HIPVs) [4]. HIPVs may defend plants by either directly repelling or deterring herbivores [5,6], or indirectly by attracting natural enemies of herbivores [7–9]. In addition to facilitating trophic and multi-trophic interactions, HIPVs are involved in information transfer between plants, which is often referred to as plant–plant communication [10]. Stem feeding on conifers induces substantial VOC emissions [11], which provide potentially strong HIPV cues to other organisms in the community [12]. However, despite such HIPV-mediated between-plant communication being documented in numerous plant species [10], earlier studies have chiefly focused on herbaceous and broadleaved woody plants, with conifers largely unexplored. Given that conifers dominate many terrestrial ecosystems and some of them are experiencing increased pressure from forest pests [13], studying it is crucial to further understanding plant communication and the scope for its use in the management of conifer forests.

As cues mediating plant communication, HIPVs may advertise the presence of herbivores, providing plants with sufficient information to pre-empt the imminent arrival of herbivores and tailor their defences. Undamaged HIPV-receiver plants may initiate and/or prime defences [14–20]. For example, plants exposed to HIPVs have been shown to upregulate defence-related genes [21,22] and increase production of phytohormones [23], proteinase inhibitors [24], terpenoids [23,25] and extrafloral nectar [26–28]. In some cases, plants exposed to HIPVs do not show immediate changes in their defence responses but respond more quickly and more strongly when they are attacked by herbivores [29]. This phenomenon is known as ‘priming’. For example, cabbage plants exposed to HIPVs were primed for stronger VOC emissions upon subsequent herbivore-attack than were unexposed plants [30]. There is a growing body of studies demonstrating the priming effects of HIPVs [23,25,29–33]. However, although conifer VOCs can act as a defence against herbivores [13], induction or priming in response to HIPVs has not earlier been documented.

Scots pine (*Pinus sylvestris*) is a dominant conifer species in the boreal forest that emits VOCs via de novo synthesized and stored pools [34]. The de novo emissions of VOCs are primarily driven by photosynthesis [35–38], which also provides carbon (C) resources for other defence-related traits or for compensatory growth to resist herbivore-damage [39]. In conifers, emissions from stored pools refer to those from specialized organs that store resin, such as resin ducts (RDs), and are related to the characteristics of those structures [40]. RDs can also channel terpenoid-containing resin to wound sites to form sticky physical barriers against pests and pathogens [40]. Hence both photosynthesis and RD characteristics are expected to play roles in conifer defence against herbivores. However, changes in photosynthesis and VOC storage structures in response to informative HIPV cues require a greater level of integration into a plant–plant communication framework. Furthermore, consideration of abiotic factors that directly impinge upon the fidelity of the HIPV cue or directly affect plant physiological responses has been largely neglected in this context.

Ground-level ozone (O_3_) is an important phytotoxic pollutant that has been increasing globally [41]. For example, current O_3_ levels in rural areas of the temperate and polar zones of the Northern Hemisphere have increased by 30 to 70% compared to 1896–1975 [42,43]. O_3_ is an important phytotoxic air pollutant that can cause altered photosynthesis, respiration, carbon allocation, stomatal functioning and VOC emissions [44,45]. O_3_ also reacts rapidly with many VOCs in the air, such as green leaf volatiles [46] and terpenes [47]. In a recent synthesis, O_3_ has been linked to biodiversity decline through its effects on key physiological traits of plants, foliar chemistry and plant–soil–microbe interactions [48]. The reactivity and phytotoxicity of O_3_ make it a significant gas with respect to between-plant communication. Several studies have shown that O_3_ disrupts HIPV-mediated between-plant communication [25,27,30,49,50], but all earlier studies concerned broadleaved plants and foliage-feeding herbivores.

The large pine weevil (*Hylobius abietis* L.) is a major pest of coniferous forests in Europe [51]. Although adult weevils also feed on the bark of adult trees, the greatest damage occurs in seedlings, on which feeding activity can easily lead to stem girdling and seedling death [52,53]. Here we use *P. sylvestris* seedlings and *H. abietis* weevils to investigate whether HIPVs can mediate between-plant communication in conifers and how elevated O_3_ affects the process. By exposing receiver plants to either undamaged or weevil-infested emitter plants under ambient or enriched O_3_ levels, we show that HIPV exposure induces and primes VOC emission, increases stomatal conductance and net photosynthesis rate, alters RD traits and decreases damage caused by *H. abietis*. We also show that elevated air pollution, represented by ozone, can affect the plant responses, but does not appear to eliminate the increased resistance of receiver plants. These findings confirm that conifers can use HIPVs as a warning cue to improve their pest resistance by enhancing photosynthesis and chemical defence, while O_3_ partially impedes the process.

## 2. Material and methods

### (a) Plants and insects

Two-year-old *P. sylvestris* seedlings were used in this study. The seedlings were removed from cold storage and transferred to a mixture of sand and peat (2 : 1) in 3 l pots. they were positioned on the roof of a building on the kuopio campus (62°53′31″ N, 27°38′20″ E) of the University of Eastern Finland and then transferred to plant growth chambers (75 cm W; 128 cm L; 130 cm H) (Weiss Bio 1300; Weiss Umwelttechnik Gmbh, Reiskirchen-Lindenstruth, Germany) (day: 20 h light (photosynthetically active radiation (PAR) 300 µmol m^−2^ s^−1^), 17°C, 60% humidity; night: 4 h dark, 13°C, 80% humidity) for experiments.

On 8 June 2020, *H. abietis* weevils were collected by individual hand picking from a fresh clear-cut area (63°11′38″ N, 30°38′72″ E) where *P. sylvestris* trees were harvested during the preceding winter. The area was government land owned and managed by Metsähallitus, and the weevil collection was done with a research permit granted to the Natural Resources Institute Finland (Luke) (MH 2491/2020). After collection, the weevils were stored at +8°C in plastic containers and provided with cut pine branches as a food source. Prior to the initiation of experiments, the weevils were starved for a 24 h period.

### (b) Experimental design

To test whether HIPVs can mediate between-plant communication in Scots pine and how elevated O_3_ affects the process, we performed two experiments. In the first experiment, we investigated the effect of HIPV exposure on VOC emissions, gas exchange parameters and weevil-induced damage of HIPV-receiver seedlings under ambient and elevated O_3_ conditions. The first experiment had three phases. In the first phase, seedlings were arranged in plant growth chambers (conditions as described above) in two parallel rows with three seedlings in each row and a 30 cm space between rows. Seedlings on the right side of the chambers were designated as emitters, while seedlings on the left were undamaged receivers, with air passing from right to left (electronic supplementary material, figure S1). Four chambers were used concurrently; in two chambers, the O_3_ levels were elevated to 80 nmol mol^−1^ (dropping to 30 nmol mol^−1^ O_3_ at night), while in two chambers, the ambient O_3_ level (approximately 15 nmol mol^−1^) was used. An 80 nmol mol^−1^ O_3_ was selected because it has been occurring in some parts of Europe and will become more frequent in the future [54,55]. The emitters from one chamber in each O_3_ regime were infested with three *H. abietis* weevils enclosed in a mesh sleeve (covering an 8 cm-long section of the main stem starting from the base). The remaining chamber in each O_3_ regime had undamaged emitters with a mesh sleeve but without weevils and acted as a control. This set-up was maintained for 5 days. After 5 days in the chambers, all receivers were taken to the laboratory for VOC collection and gas exchange analysis (this is considered time 0 h). In addition, one or two emitters from each chamber were randomly selected for VOC collection in the laboratory. As soon as the measurement of the first phase was completed, all emitters were taken away from the chambers. In the second phase, each receiver was then infested with three weevils enclosed in a mesh sleeve (covering the main stem in the same way as for the emitters) and placed back into the corresponding chambers. At 18 h after infestation, VOC collection, gas exchange analysis and damage area determination were conducted on all receivers in a laboratory (this is considered time 18 h). *Hylobius abietis* weevils were removed before these measurements and were then put back on the corresponding receivers when measurements ended. In the third phase, we repeated the same procedure (e.g. 18 h infestation) described in the second phase, which is considered time 36 h. Between measurement intervals, the receivers were kept in chambers with O_3_ regimes maintained. We ran the experiment four times from 25 June to 23 July 2020 with chamber rotation each time.

To investigate needle anatomy following exposure of seedlings to the same treatments described above, we conducted a second experiment. Four emitters and four receivers were placed in each of four chambers on 14 August 2020. Aside from having an additional emitter and receiver plant, the seedlings were arranged and treated in the same way as for phase one above. After 5 days of exposure to the different treatments, the needles were sampled from each receiver for anatomical observation. To reduce the possibility of a chamber effect, the chambers were rotated once on the third day during these 5 days. The seedling growth had stopped in the period between the two experiments.

### (c) Volatile organic compound collection and analysis

Seedlings were sampled in laboratory conditions at room temperature (20–22°C) with a PAR level of approximately 250 µmol m^−2^ s^−1^. The whole above-ground part of each seedling was carefully enclosed in a polyethylene terephthalate (PET) cooking bag (LOOK, Fredman Group, Espoo, Finland). The bag size was 35 × 43 cm, which was conditioned by pre-heating for 1 h at 120°C. Purified inlet air generated from a zero-air generator (Aadco Instruments, 747–30, Cleves, OH, USA) was humidified and then introduced into the bags at a flow rate of 400 ml min^−1^. After 30 min of flushing air through the bag, VOCs were collected for 10 min into a cleaned stainless steel tube (Perkin Elmer, Boston, MA, USA) filled with adsorbents (Tenax TA and Carbopack B, 100 mg of each, mesh 60/80, Supelco, Bellefonte, PA, USA) positioned at the outlet of the bag. The stainless steel tube was connected via clean silicone tubes to a vacuum pump (D-79112, KNF, Germany), which pulled the air through the stainless steel tube with a flow rate of 200 ml min^−1^. Inlet and outlet airflows were calibrated with a flow meter (mini-Buck calibrator, AP Buck Inc., Orlando, FL, USA). A higher inlet air flow was used to create overpressure and to prevent outside VOCs from leaking into the bag. An empty bag (blank) sample was also collected in each measurement round so that background contamination could be identified and removed. The stainless steel tubes were sealed with brass caps immediately after sampling, refrigerated (+5°C) and analysed within 5 days.

Analysis of VOCs collected was performed by gas chromatography-mass spectrometry (GC-MS) (Agilent 7890A GC and 5975C VL MSD; New York, USA). Trapped compounds were desorbed with a thermal desorption unit (TD-100; Markes International Ltd., Llantrisant, UK) at 300°C for 10 min and then cryofocused at −10°C. The compounds were then transferred in split mode to an HP-5MS UI capillary column (60 m × 0.25 mm; film thickness 0.25 µm) with helium as a carrier gas. The oven temperature was held at 40°C for 1 min, then programmed to increase by 5°C min^−1^ to 125°C and then by 10°C min^−1^ to 260°C with a column flow of 1.2 ml min^−1^. Mass spectra were obtained by a scanning from 33 to 400 m z^−1^. Compounds were identified by comparing their mass spectra with those of compounds in the Wiley library and with pure standards. The compounds other than those included in the standards were quantified by comparing to other compounds with similar chemical structures. For example, the compound α-pinene was used as a reference for non-oxygenated monoterpenes (MT-no), 1,8-cineole for oxygenated monoterpenes (MT-ox) and β-caryophyllene for sesquiterpenes (SQTs).

VOC emission rates were calculated as ng g^−1^ dry mass (DM) h^−1^ using equation (2.1):

2.1 E=(F×(C2–C1))/M,

where *E* = VOC emission rate (ng g^−1^ DM h^−1^), *F* = flow rate of inlet air (l h^−1^), C2 = VOC concentration in outlet air (ng l^−1^), C1 = VOC concentration in inlet air (ng l^−1^, considered to be 0 because inlet air was filtered, and quantities of VOCs determined from the empty PET bag samples were subtracted from emissions), *M* = dry mass of the above-ground part of the seedlings (g).

### (d) Gas exchange analysis

Once the VOC collection was completed, receivers were measured for gas exchange (stomatal conductance and net photosynthesis rate) from previous- and current-year main shoots. Measurements were done using a LiCOR 6400 XT equipped with an opaque conifer chamber (Licor 6400-22; Licor) and a red-green-blue light source (Licor 6400-18; Licor) at saturating light level (1500 µmol m^−2^ s^−1^ determined from light saturation curves), CO_2_ concentration of 410 ppm and air temperature of 25°C.

Gas exchange was related to the total needle area (inside the conifer chamber) as described by equation (2.2) [56]:

2.2 At=((4.2235×L−15.6835)×N)/100,

where At = needle area (cm^2^), *L* = average needle length (mm) and *N* = needle number.

### (e) Damage area determination

After gas exchange analysis at 18 and 36 h after infestation, the area of bark removed by the weevils was measured. This was done by drawing around the sites of damage onto a plain sheet of paper with a square of known area (1 cm^2^) on the page. We then photographed the page and the photographs were analysed using the ImageJ programme (v. 1.47).

### (f) Microscopy

Five green and matured needles were carefully detached from the needle base by tweezers from the current-year main shoot of each receiver. An approximately 1.5 mm segment was sectioned from the middle of each needle in cold prefixative containing 2.5% glutaraldehyde (Electron Microscopy Sciences, Hatfield, Pennsylvania, USA) in 0.075 mol l^–1^ cacodylate buffer, pH 7.2. The next day, samples were processed with a Lynx Microscopy Tissue Processor (Reichert-Jung Optische Verke AG, Wien, Austria) as follows: 0.075 mol l^–1^ cacodylate buffer 2 × 15 min (+4°C), 1% osmiumtetroxide (Electron Microscopy Sciences) in 0.075 mol l^–1^ cacodylate buffer for 6 h (+4°C), 0.075 mol l^–1^ cacodylate buffer 3 × 10 min (+4°C), increasing ethanol series (50%, 70%, 94% and 100%) each 2 × 10 min (+4°C), propylene oxide (Sigma-Aldrich, Steinheim, Germany) 2 × 15 min (+20°C), propylene oxide : epon (Ladd LX112, Burlington, Vermont, USA) 3 : 1 for 1 h (+20°C), propylene oxide : epon 1 : 1 for 1 h (+20°C), propylene oxide:epon 1 : 3 for 2 h (+20°C) and pure epon overnight (+20°C). The sections were embedded in Ladd's epon in flat embedding moulds made of silicon (Electron Microscopy Sciences) and polymerized first at +37°C for 24 h and then at +60°C for 72 h. Semi-thin (1.0 µm) sections of three needles per seedling were stained by toluidine blue solution (1 ml 1% toluidine blue and 20 ml 2.5% sodium bicarbonate) for 10 min for light microscopy (LM). The sections were photographed by light microscope (Carl Zeiss AxioImager M2, camera Axiocam MRc, Jena, Germany) using 5 × and 40 × objectives. The following parameters were determined from the digital images using tools of the ImageJ programme (v. 1.47): needle cross-sectional area, RD number (per needle section and per unit of needle area), RD area (per needle section and mean area), proportion of RD area per needle cross-sectional area, epithelial cell (EC) number (per needle section and per RD), EC area (per RD, per needle section and mean cell area), proportion of ECs area per RD area and the second layer EC number per RD.

### (g) Statistics

All data were tested with linear mixed models (LMMs) by using IBM SPSS Statistics 27. For net photosynthesis rate, stomatal conductance and damage area-based increases in VOC emission rate (calculated as the emission at 18 and 36 h after infestation minus the emission before infestation divided by the damage area recorded at 18 and 36 h after infestation), the data were analysed with HIPV exposure, O_3_ treatment and post-infestation time as fixed factors and the replicate number as a random factor. For VOC emission rates from receivers, data from different post-infestation times were analysed separately with HIPV exposure and O_3_ treatment as fixed factors and replicate number as a random factor. For data of VOC emission rate from emitters, weevil feeding and O_3_ treatment were fixed factors and replicate number was a random factor. Anatomical features were analysed with HIPV exposure and O_3_ as fixed factors and plant identity as a random factor. *p*-values ≤ 0.05 for main effects were considered statistically significant. All interactions with *p*-values ≤ 0.1 were considered statistically significant and further tested for simple main effects (SME; i.e. post hoc tests for interactions) with Bonferroni corrections, where the SMEs with *p* ≤ 0.05 were used for interpreting the interactions, similar to in other ecological experiments [57–59]. For any statistical analyses where the assumption of normality of residuals was violated, log (*x* + 1) transformed data were used.

## 3. Results

### (a) Exposure to herbivore-induced plant volatiles reduces subsequent damage by herbivores in receiver plants

**Figure 1.**
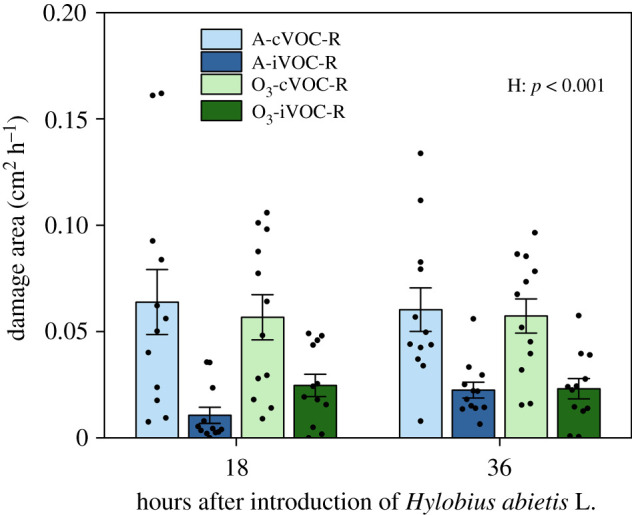
Damage area (mean ± s.e., 12 plants from four replicates) to receiver plants per hour. Measurements were taken at 18 and 36 h after infestation with the damage areas expressed in cm^2^ h^−1^ for the periods 0–18 h and 18–36 h. Receivers (R) were exposed to either undamaged (cVOC) or weevil-infested emitters (iVOC) under ambient O_3_ (A) or enriched O_3_ levels (O_3_). *p*-values of main effects (*p* ≤ 0.05) and interactions (*p* ≤ 0.1) from LMM are shown. Treatment abbreviations: H = HIPV exposure. (Online version in colour.)

The stem damage was significantly less on HIPV-exposed plants than on plants exposed to undamaged neighbours (the main effect of HIPV exposure (H), *p* < 0.001) (figure 1; electronic supplementary material, table S1). Since there were no two-way or three-way interactions among O_3_ treatment (O), H and time post-infestation (*p* > 0.1) (electronic supplementary material, table S1), this result indicates that HIPV exposure improves *P. sylvestris* resistance to herbivores in a process that is not significantly affected by elevated O_3_.

### (b) Exposure to herbivore-induced plant volatiles induces the emission of volatile organic compounds in receiver plants

**Figure 2.**
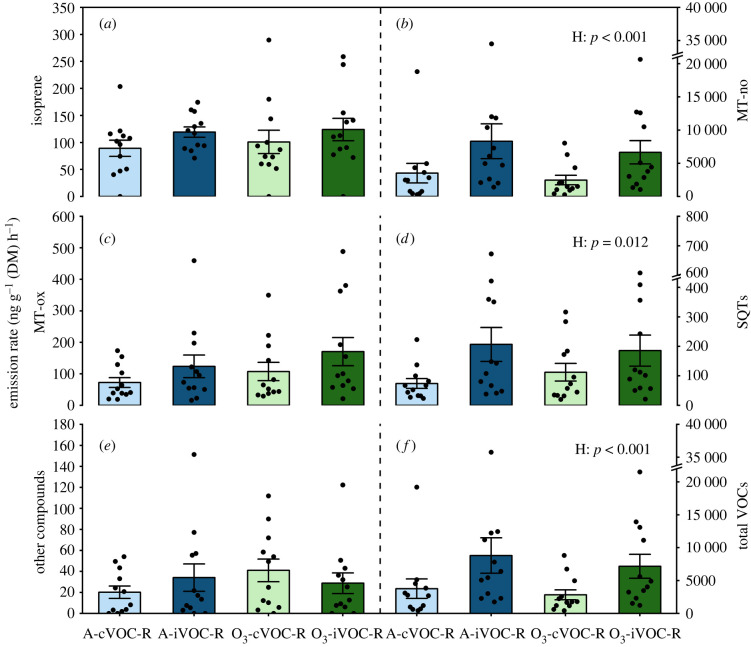
Total emission rates (mean ± s.e., 12 plants from four replicates) of isoprene (*a*), MT-no (*b*), MT-ox (*c*), SQTs (*d*), other compounds (*e*) and all VOCs (*f*) from receivers before weevil infestation. Receivers (R) were exposed to either undamaged (cVOC) or weevil-infested emitters (iVOC) under ambient O_3_ (A) or enriched O_3_ levels (O_3_). *p*-values of main effects (*p* ≤ 0.05) and interactions (*p* ≤ 0.1) from LMM are shown. Treatment abbreviations: H = HIPVs exposure. (Online version in colour.)

In total, 43 compounds were emitted from the *P. sylvestris* seedlings before weevil infestation, including isoprene, MT-no, MT-ox, SQTs and other compounds (electronic supplementary material, table S2). Analysis with LMMs showed that HIPV exposure for 5 days increased emissions of total MT-no by 150% (*p* < 0.001), total SQTs by 138% (*p* = 0.012) and total VOCs by 142% (*p* < 0.001) (figure 2; electronic supplementary material, table S2). In total, 15 MT-no compounds, two MT-ox compounds and eight SQT compounds were found to increase significantly in response to HIPV exposure (*p* < 0.05) (electronic supplementary material, table S2). No interaction between O_3_ and HIPV exposure was found for emissions of any of the six VOC groups or individual compounds (*p* > 0.1, except longifolene *p* = 0.099) (electronic supplementary material, table S2), thus confirming that HIPV exposure induces the emission of some VOCs in a process that is not affected by elevated O_3_.

### (c) Herbivore-induced plant volatile exposure primes receiver plants for enhanced volatile organic compound emission, but elevated O_3_ suppresses the effect

**Figure 3.**
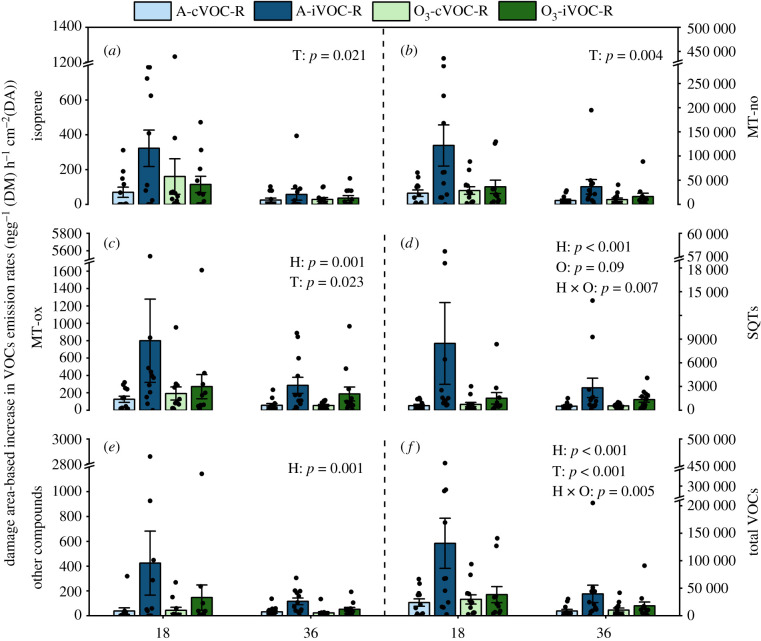
Damage area-based increases in VOC emission (mean ± s.e., 11 or 12 plants from four replicates) of isoprene (*a*), MT-no (*b*), MT-ox (*c*), SQTs (*d*), other compounds (*e*) and all VOCs (*f*) in receivers, which are calculated as the emission at 18 and 36 h after infestation minus the emission before infestation divided by the damage area recorded at 18 and 36 h after infestation. Receivers (R) were exposed to either undamaged (cVOC) or weevil-infested emitter plants (iVOC) under ambient O_3_ (A) or enriched O_3_ levels (O_3_). *p*-values of main effects (*p* ≤ 0.05) and interactions (*p* ≤ 0.1) from LMM are shown. Treatment abbreviations: H = HIPVs exposure, O = O_3_ treatment and T = post-infestation time. (Online version in colour.)

Damage area-based increases in VOC emission were used to determine the priming of VOC emission. LMMs revealed a significant main effect of HIPV exposure for total MT-ox, total other compounds and 15 individual compounds (*p* < 0.05), and there were no interactive effects of O_3_ and HIPV exposure for these compounds (*p* > 0.1) (figure 3; electronic supplementary material, tables S3 and S4). However, for total VOCs and 11 individual compounds interactions between O_3_ and HIPV exposure indicated that HIPV exposure only primed plants for VOC emission under ambient O_3_ levels, while for total SQTs and three individual compounds, the extent of priming was more pronounced under ambient O_3_ than elevated O_3_ (H+ versus H- in O-, H+ versus H- in O+) (electronic supplementary material, tables S4 and S5). These results reveal a clear priming of VOC emission in plants exposed to HIPVs but elevated O_3_ suppresses the effect for some compounds.

### (d) Herbivore-induced plant volatile exposure increases net photosynthesis rate and stomatal conductance of receiver plants, but elevated O_3_ disrupts the process

**Figure 4.**
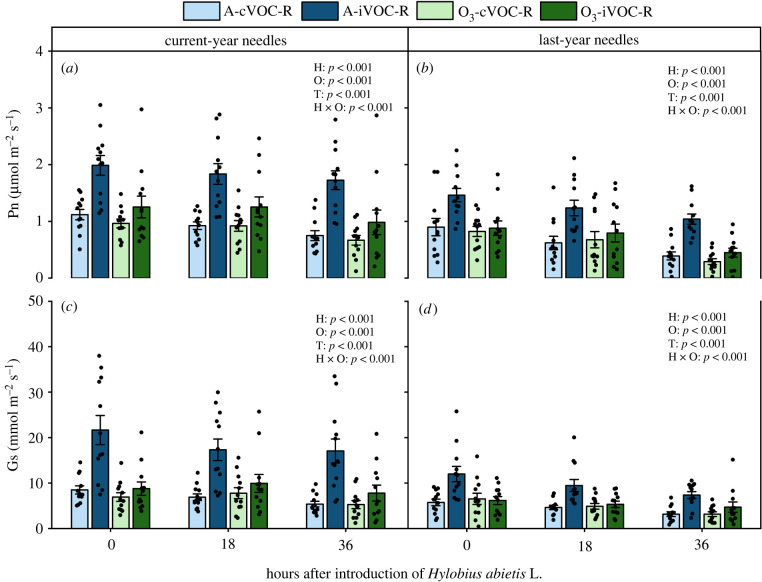
Net photosynthesis rate (Pn) (*a*,*b*) and stomatal conductance (Gs) (*c*,*d*) of current-year and last-year needles from receivers (mean ± s.e., 12 plants from four replicates). Receivers (R) were exposed to either undamaged (cVOC) or weevil-infested emitter plants (iVOC) under ambient O_3_ (A) or enriched O_3_ levels (O_3_). Pn and Gs were measured at three time points, before infestation listed as time 0, and at 18 and 36 h post-infestation with three weevils. *p*-values of main effects (*p* ≤ 0.05) and interactions (*p* ≤ 0.1) from LMMs are shown. Treatment abbreviations: H = HIPVs exposure, O = O_3_ treatment and T = post-infestation time. (Online version in colour.)

The net photosynthesis rate of current-year needles was significantly affected by an interactive effect of O × H (*p* < 0.001) (figure 4*a*; electronic supplementary material, table S6). On the basis of tests for SME, HIPV exposure increased net photosynthesis rate more under ambient O_3_ (99%, H+ versus H- in O-, *p* < 0.001) than under enriched O_3_ levels (37%, H+ versus H- in O+, *p* = 0.004) (electronic supplementary material, table S7). A similar trend was observed for stomatal conductance (figure 4*c*; electronic supplementary material, tables S6 and S7). In the case of the previous-year needles, there were statistically significant interactive effects of O × H on net photosynthesis rate and stomatal conductance with HIPV exposure significantly increasing them both under ambient O_3_, but not elevated O_3_ (O × H interaction; net photosynthesis rate, 96%, H+ versus H- in O-, *p* < 0.001; stomatal conductance, 113%, H+ versus H- in O-, *p* < 0.001) (figure 4*b,d*; electronic supplementary material, tables S6 and S7). These results suggested that exposure to HIPVs increased net photosynthesis rate and stomatal conductance under ambient O_3_ level, whereas HIPV-induced increases in net photosynthesis rate and stomatal conductance were reduced (for current-year needles) or eliminated (for last-year needles) by elevated O_3_.

### (e) Herbivore-induced plant volatile exposure influences anatomical features of receiver plants under ambient O_3_ conditions

**Figure 5.**
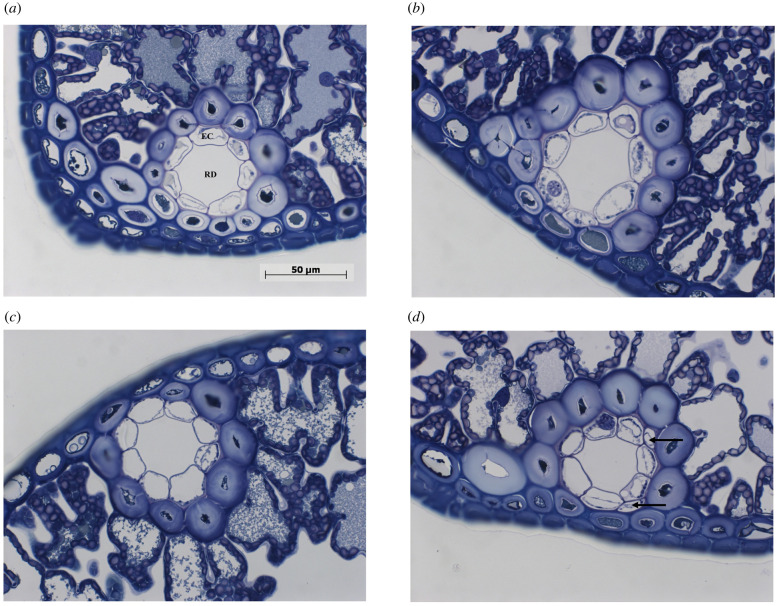
Representative light micrographs of RDs containing EC of current-year needles of receivers exposed to non-infested emitters under ambient O_3_ levels (*a*), weevil-infested emitters under ambient O_3_ levels (*b*), non-infested emitters under enriched O_3_ levels (*c*), and weevil-infested emitters under enriched O_3_ levels (*d*). Note black arrows indicate the second layer ECs. (Online version in colour.)

Light micrographs of RDs containing ECs are shown in figure 5. There was an interactive effect of O × H on EC mean area with HIPV exposure significantly increasing the area under ambient O_3_ (O × H interaction, *p* = 0.093; 42%, H+ versus H- in O-, *p* = 0.013) but not under elevated O_3_ (electronic supplementary material, tables S8 and S9). The number of second layer ECs per RD was marginally increased in response to HIPV exposure (H main effect, *p* = 0.065), but no O × H interaction was found (electronic supplementary material, table S8). This indicates that HIPV exposure alters EC features, but the effect on EC mean area is only observed under ambient O_3_ conditions.

## 4. Discussion

**Figure 6.**
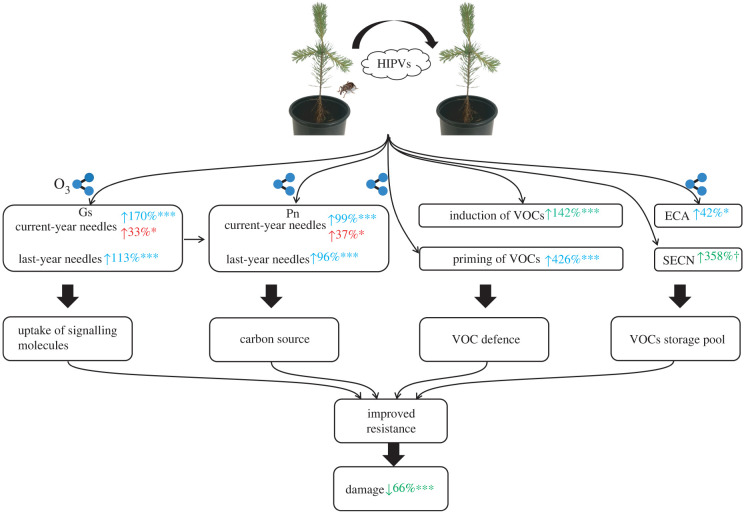
A comprehensive scheme of between-plant interactions in Scots pine. Main effects of HIPV exposure with *p* ≤ 0.1 are shown as green when there are no interactions. SME of HIPV exposure with *p* ≤ 0.05 under ambient and elevated O_3_ levels are shown as blue and red, respectively, when there are interactions. The percentage values are the changes caused by HIPV exposure compared to with non-HIPV exposure. The direction of HIPV exposure effects is indicated by the arrow direction in front of the percentage values (increase—upwards arrow or decrease—downwards arrow). The percentage values following the VOCs are calculated based on the results of total VOC groupings. Significance is denoted by ^†^*p* ≤ 0.1, *p ≤ 0.05, **p ≤ 0.01 and ***p ≤ 0.001. The icon of an O_3_ molecule indicates the response to HIPV exposure was affected by elevated O_3_ level, i.e. there is an interaction of O_3_ treatment and HIPV exposure. Abbreviations in the figure: biogenic VOCs, HIPVs, net photosynthesis rate (Pn), stomatal conductance (Gs), epithelial cell mean area (ECA) and second layer EC number per resin duct (SECN). (Online version in colour.)

In this paper, we present evidence that pre-exposure of *P. sylvestris* to HIPVs from conspecific neighbours can reduce the subsequent damage caused by *H. abietis*, induce and prime VOC emissions, increase net photosynthesis rate and stomatal conductance, and alter RD features. These strong responses are indicative of a comprehensive and biologically relevant metabolic shift involving the preparation and deployment of plant defences. Importantly, some of these responses were disturbed by elevated O_3_, but the overall effect on plant resistance to herbivores was not significantly affected (figure 6). The results of the study are bifurcate, emphasizing a highly promising study framework for plant–plant interactions and the importance of considering abiotic plant stresses on signalling processes mediated via an external medium.

A key observation in the present study was reduced herbivore-damage to Scots pine saplings that had been exposed to HIPVs, which is indicative of a gain in resistance. The underlying mechanism for this ecological effect is probably related to induced or primed defence responses [15,23,60]. VOCs are an important component of pine defence; they are toxic to bark beetles at high vapour concentrations, which has been shown to inhibit their feeding activity [61,62]. Here we observed that *P. sylvestris* exposed to HIPVs exhibited a direct induction of VOC emissions before exposure to *H. abietis*. Such a response would be expected to reduce the potential for imminent damage by herbivores. Upon feeding by *H. abietis*, *P. sylvestris* pre-exposed to HIPVs showed enhanced damage area-based increases in emissions of some VOC groups and individual compounds. This observation reveals a priming of VOC emissions in response to HIPV exposure. Our study clearly demonstrates between-plant communication to mediate induction and priming of VOC emissions as a possible defence in an important conifer species. Further studies, for example testing the chemoreception and behaviour of *H. abietis* by using synthetic VOC blends will help to confirm these findings. To better understand the basis of the plant response to HIPV exposure, we measured stomatal conductance and net photosynthesis rate, two important plant physiological indices. Exposure of *P. sylvestris* to HIPVs increased stomatal conductance. This response suggests a greater uptake of signalling molecules by receivers. Therefore, the receivers are expected to gain more information about their environment and that can be used to gauge potential threats. Additionally, increased stomatal conductance may contribute to the increase in net photosynthesis rate because greater stomatal conductance enables more CO_2_ to be taken up by plants through stomata as substrates for photosynthesis. As expected, greater net photosynthesis rate was found in *P. sylvestris* exposed to HIPVs suggesting that greater C resources were available for the production of VOC-based chemical defence and leading to less damage being caused by *H. abietis*.

In addition to the production of defensive VOCs, a greater photosynthesis rate is expected to provide more resources for other C-based defensive chemicals, physical barriers and compensatory growth [4,60,63]. A physical structure related to the defensive chemistry of Scots pine is the RD, tube-like structures formed by secretory ECs that produce and exude terpenoid resin into the lumen [64]. Therefore, the traits of ECs are likely to affect VOC synthesis, and alterations to those cells could account for changes to VOC emissions as part of a systemic response. RDs are normally composed of one layer of ECs [65]. Interestingly, the structure of RDs was investigated in the present study and showed that *P. sylvestris* exposed to HIPVs tended to have a greater number of second layer ECs per RD. Furthermore, the EC mean area was also increased in response to HIPVs. These induced alterations to ECs are expected to facilitate greater terpenoid synthesis for VOC-based chemical defence. A greater amount of terpenoids synthesized and stored in RDs will also enable terpenoid resin exudation to seal wounds and prevent the entry of herbivores by forming a crystallized resin barrier after damage [66].

Volatile-mediated interactions are potentially vulnerable to abiotic factors such as air pollution, but under elevated O_3_ conditions Scots pine saplings that received HIPVs also gained resistance to herbivores. This suggests that the HIPV-mediated induction of VOC-based defence persists under O_3_ stress; however, elevated O_3_ suppressed the priming of some VOC emissions, suggesting that the receiver plant response was not identical to that at ambient O_3_ levels. Elevated O_3_ can impede VOC-mediated plant–plant communication at three junctures [67], which are first at the emitter, which may emit an altered VOC blend in response to O_3_ stress [68–70], second in the air between emitter and receiver, where degradation of signalling VOCs may occur [27,44], and third at the receiver, where the ability of plants to detect a VOC signal may be altered [67]. Since elevated O_3_ had negligible effects on VOC emissions from the emitter plants (electronic supplementary material, tables S10 and S11) and HIPV-mediated induction of VOC emissions still occurred, it seems that effects on the emission and transport of the signalling VOCs can be excluded. Therefore, it is most likely that elevated O_3_ interferes with the process at the receiver plant through the reduction or elimination of HIPV-induced increases in stomatal conductance. This would potentially lead to decreases in HIPV-induced uptake of a VOC signal. In line with the effects on stomatal conductance, HIPV-induced increase in net photosynthesis rate was also reduced in response to O_3_. A potential explanation for these results is that greater stomatal conductance facilitates a greater O_3_ flux into the plant (i.e. greater O_3_ toxicity) [71], which is known to induce stomatal closure and sluggishness. This direct response to O_3_ would probably reduce the extent of VOC signalling. In this study, besides impairing a receiver's ability to receive a VOC signal, elevated O_3_ might interfere with between-plant communication via an alternative route. The O_3_-induced reduction in net photosynthesis rate is also likely to have reduced the HIPV-induced increases in C resources that might be used for VOC synthesis. Under O_3_ stress, HIPV-induced increases in the number of second layer ECs per RD was not affected, but the increases in EC mean area were reduced. Based on our results, there could be two potential reasons for this O_3_ effect: (i) receivers may not suitably tune their defence strategy according to the level of herbivore risk owing to elevated O_3_ impairing the receiver's ability to detect a VOC signal and respond to potential risks; and (ii) less C is available to increase EC mean area owing to elevated O_3_ reducing HIPV-induced increases in net photosynthesis rate.

In conclusion, we propose that HIPVs can mediate between-plant communication in an ecologically and economically important conifer species, *P. sylvestris*, and as a result decrease the susceptibility of receivers to subsequent herbivore feeding. During this process, VOC emissions, which represent possible defences against *H. abietis*, are induced and primed by HIPV exposure. In addition, increases in stomatal conductance and net photosynthesis rate and altered RD traits owing to HIPV exposure may contribute to the enhanced resistance. Enhanced O_3_ levels interrupt a few of the processes activated by HIPVs, but do not significantly affect the final defence outcome (as indicated by damage area). Our data support the potential application of between-plant communication to defence against insect herbivory in conifers and reveals how the defence strategy is adjusted for adapting to O_3_ pollution.

## Data Availability

The data are provided in the electronic supplementary material [72].
